# A case-control study on preauricular congenital fistula surgery outcomes by Propensity Score Matching (PSM) analysis

**DOI:** 10.1016/j.bjorl.2025.101729

**Published:** 2025-11-26

**Authors:** Jibing Sun, Tingting Wang, Dong Chen, Yanyan Mao, Zhaomin Fan, Yuechen Han

**Affiliations:** Shandong Provincial ENT Hospital, Shandong University, Department of Otoplasty, Jinan City, Shandong Province, China

**Keywords:** Propensity score matching, Stage I surgery, Congenital preauricular fistula, Localized infection stage, The quiescent inflammatory stage

## Abstract

**Objective:**

To explore the propensity matching score to compare the clinical efficacy of infection localized and inflammatory quiescent stage I surgery in the treatment of congenital preauricular fistula.

**Methods:**

120 patients with congenital preauricular fistula treated in our hospital from January 2022 to August 2023 were selected. The patients were divided into infection limited group (n = 63) and inflammation rest group (n = 57) according to the mode of operation. The two groups were matched at 1:1 with Propensity Score Matching (PSM) method, and finally included in infection limitation group and inflammation rest group (n = 50). Patients in both groups were treated with stage I operation.

**Results:**

Before PSM, there were significant differences between the two groups in terms of gender, age, BMI, and the number of patients with previous infection requiring incision and drainage (p < 0.05). After PSM, there were no statistically significant differences in general information between the two groups (p > 0.05). There was no significant difference in healing, incidence of postoperative complications and recurrence rate between the two groups (p > 0.05).

**Conclusion:**

There is no significant difference in the clinical efficacy comparison of surgical treatment for congenital preauricular fistula between the infection-localized phase and inflammation-quiescent stage I surgery with PSM.

**Level of Evidence:**

Level 3.

## Highlights

• PSM method used to match infection-localized and inflammation-quiescent groups.
• No significant differences in healing or complications after stage I surgery.
• PSM successfully balanced patient demographics for accurate clinical comparison.

## Introduction

Congenital preauricular fistula is a common congenital external ear malformation, which is usually caused by incomplete fusion of the first and second branchial arches or incomplete closure of the first branchial groove during embryonic development. It is manifested as a blind-ended small tube that is often located at the anterior skin of the helical root, but can also be found above the helical root, tragus, and lobule skin.^1^^,^^2^ Once the fistula opening of the congenital preauricular fistula is blocked by desquamated squamous epithelium or secretions, secondary bacterial infection and abscess formation may occur, requiring surgical excision.

However, during the infection of the preauricular fistula, there is inflammatory hyperplasia of the surrounding tissue and multiple branches of the fistula, making the infection spread with unclear boundaries. The risk of residual sinus tissue leading to recurrent infections of the preauricular fistula exists. Therefore, the traditional concept suggests that surgical excision should be performed after controlling the infection and achieving stability.^3^^,^^4^ For some patients with recurrent preauricular fistula infections, persistent non-healing of the abscess after incision and drainage, and some patients experiencing repeated purulent infections before the infection stabilizes, multiple incisions and drainages may be performed, resulting in unsatisfactory outcomes and significant physical and psychological distress for the patients.

Propensity Score Matching (PSM) is a statistical method used to address selection biases in observational studies.^5^^,^^6^ By using a score to replace multiple covariates, it balances the distribution of covariates between the treatment group and the control group, reducing selection biases.^7^ The incidence of congenital preauricular fistula is approximately 1.2%, with a male-to-female ratio of 1:1.7. The ratio of unilateral to bilateral cases is 4:1, and the infection rate is about 20%.^8^ Some patients with infected congenital preauricular fistula experience prolonged non-healing wounds and require frequent dressing changes, causing significant discomfort. The main reason for not performing surgery during the infection phase is the concern that the infected fistula might spread, making complete excision of the fistula tract challenging. Therefore, for patients with persistent infection and controlled inflammation in the fistula tract, early surgical intervention can significantly shorten the disease course and alleviate patient suffering.^9^

In this study, the collected patient data would be processed with PSM to compare the clinical efficacy of surgical treatment for congenital preauricular fistula between the localized infection stage and the quiescent inflammatory stage, providing references for clinical surgery.

## Methods

### Research objects

120 patients with elective surgery for congenital preauricular fistula, admitted to our hospital from January 2022 to August 2023, were selected. The patients were divided into two groups based on the surgical approach: the infection-localized group (n = 63) and the inflammation-quiescent group (n = 57). The limited period of congenital preauricular fistula infection is characterized by limited redness and swelling around the fistula, obvious local tenderness, no fluctuations, and no systemic symptoms or mild; in the static period of inflammation, phase I, the skin around the fistula returns to normal color and texture, without redness and swelling, pain, no secretions overflow, and a relatively stable state of no inflammatory activity.^10^ Inclusion criteria were as follows: (1) Clinical diagnosis of congenital preauricular fistula; (2) Patients with unilateral occurrence; (3) Patients without surgical contraindications. Exclusion criteria were as follows: (1) Patients with preauricular fistula infection involving cartilage and concurrent auricular chondritis; (2) Patients with elevated body temperature and severe systemic symptoms caused by infection; (3) Patients with concomitant otitis media or other ear diseases; (4) Patients with severe heart, liver, or kidney diseases; (5) Patients with impaired mental status.

This study has been approved by the Ethics Committee of Hospital. Informed consent was waived for this retrospective study due to the exclusive use of de-identified patient data, which posed no potential harm or impact on patient care.

### Treatment methods

During the surgery, all patients received intravenous antibiotic prophylaxis or antimicrobial treatment. The choice of anesthesia was based on the patient's age and psychological condition, with options including local or general anesthesia. Prior to the surgery, routine preparation of the ear area was performed, and anesthesia was administered along with standard disinfection. Different surgical approaches were used for the infection-localized group and the inflammation-quiescent group.

**Fig. 1:**
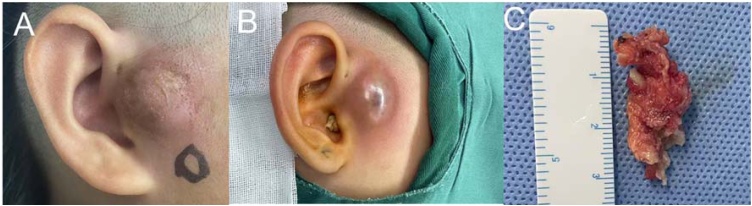
Elective surgery for congenital preauricular fistula in period of congenital preauricular fistula infection. (A) Illustrates the preoperative site, showcasing evident diseased areas on the skin surrounding the ears, characterized by irregular texture and color changes; (B) Depicts the initial stage of surgical preparation, marked by an inflammatory phase; (C) Displays the surgical resection segment, showcasing irregularly shaped excised tissue, with the size of the excised tissue clearly observable, measurable, and recordable.

For the infection-localized group, a double-ellipse incision was made, extending downward around the sinus opening. Another ellipse incision was made at the site of anterior granulation tissue or scar formation, and careful dissection of the fistula tissue was performed along the incision from the fistula opening. Microscopic techniques were used to magnify the surgical field for precise observation of the fistula tract. Sharp and blunt dissection was carried out around the fistula tract, removing the corresponding part of the auricular cartilage or scar tissue if adhesion occurred. The fistula tract and blind end were completely excised until normal surrounding tissue was reached, including the temporalis fascia and parotid fascia surfaces. The other incision was made downward along the granulation tissue, and after excision of the fascia tract and granulation tissue, a subcutaneous tunnel was formed, preserving the skin between the two incisions as much as possible. The surgical cavity was closed with 3‒0 absorbable sutures, and the two incisions were separately sutured with 5‒0 absorbable sutures. An example diagram related to the surgical content was shown in Fig. 1.

**Fig. 2:**
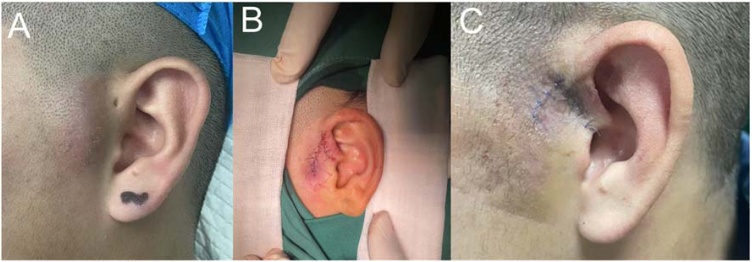
Elective surgery for congenital preauricular fistula in static period of inflammation, phase I. (A) Illustrates the preoperative condition, displaying the skin condition around the patient's ears with visible pigmentation or marks in the pre-auricular area; (B) Showcases the intraoperative suturing scene, revealing the suturing of the ear wound with the internal tissue exposed and the sutures in good condition; (C) Presents the appearance of the scar three days after surgery. The resulting scar encircling the ears displays a deeper color compared to the surrounding skin, with postoperative traces such as scabs visible on the surface, indicating a good recovery.

For the inflammation-quiescent group, a longitudinal incision was made along the fistula opening. If necessary, the incision could be extended to the affected area for patients with multiple branches or deep fistula tracts. Blunt dissection technique was used to open the skin and surrounding tissue and separate the fistula tract tissue. Careful dissection of the surrounding tissue along the fistula tract was performed until reaching the blind end of the fistula. During the dissection process, the entire fistula cyst and its branches, as well as scar and granulation tissue, were removed as much as possible while ensuring that the fistula tract was not cut. Probing was performed if necessary. For patients with fistula tracts penetrating the auricular cartilage, the attached cartilage at the site of the fistula tract was also excised. The surgical cavity was then irrigated, and electrocautery was used for hemostasis. After thorough hemostasis, the surgical site was flushed with saline solution and sutured. The sutures were removed postoperatively after 7 days, depending on the recovery status. Postoperative pathology confirmed the diagnosis of preauricular fistula with surrounding scar tissue or granulation tissue. An example diagram related to the surgical content was shown in Fig. 2.

### Observational indicators

1) Comparison of general characteristics before and after PSM in the two groups.
2) The postoperative healing was compared between the two groups. Including: hospitalization time, inflammation improvement time, scar score, the number of cases of stage Ⅰ healing, the number of cases of stage Ⅱ healing, scar score using the modified Vancouver scar rating scale for evaluation,^11^ including scar length, scar type, scar color and other 8 items, a total of 15 points, the higher the score, the more serious the scar. The standard of stage I healing: the wound healed well, there was no infection or scar, and the standard of stage II healing: there was mild infection or scar in the incision.
3) Comparison of postoperative complication rates and recurrence rates between the two groups. Complications include auricular deformity and incision infection.

### Sample size

Sample size was calculated using PASS (NCSS Ltd., Keswell, Utah, USA). According to previous pre-study on congenital preauricular fistula, the recurrence rate in the infected limited group was 1.19%, and that in the Inflammatory quiescent group was 2.61%, with a difference of 1.42% The two-sided significance threshold (α-value) was set to 5%, and the power was 80%. Using a two-sided test, at least 100 patients were needed to detect the difference between the two groups.

### Statistical analysis

The collected experimental data were analyzed by SPSS 27.0. Calculate data were expressed as [n (%)] and Chi-Square test was used. Normally distributed measurement data were expressed as mean ± SD. If the data was not normal distribution, the variable was transformed to normal distribution for statistical analysis using *t*-tests.

## Result

### Comparison of general data before and after propensity matching score between the two groups

PSM was performed in a 1:1 ratio to match the two groups, considering such variables as gender, age, Body Mass Index (BMI), number of initial occurrences, and previous cases of incision and drainage for infection. Both groups underwent primary surgical treatment performed by the same surgeon. Before the score of propensity matching, there were significant differences in sex, age, BMI and the number of patients with previous infection incision and drainage between the two groups (p < 0.05). After the tendency matching score, there was no significant difference in the general data between the two groups (p > 0.05), as showed in Table 1.

**Table 1:** Comparison of general data before and after propensity matching score between the two groups.

	Before PSM				After PSM			
General information	Infected limited group (n = 63)	Inflammatory quiescent group (n = 57)	t/χ^2^	p-value	Infected limited group (n = 50)	Inflammatory quiescent group (n = 50)	t/χ^2^	p-value
Gender male (female)	41 (22)	24 (33)	6.362	0.042	28 (22)	24 (26)	0.641	0.726
Age (years)	21.16 ± 5.16	25.16 ± 4.78	4.021	0.000	25.14 ± 3.52	25.17 ± 4.11	0.039	0.969
BMI (kg/m^2^)	22.34 ± 3.16	23.49 ± 2.16	2.124	0.036	22.31 ± 2.97	22.48 ± 2.77	0.296	0.768
Number of first-time developers	34	29	0.115	0.944	28	27	0.040	0.980
Number of patients with previous infections incised and drained	20	7	6.502	0.039	9	7	0.298	0.862

### Comparison of postoperative healing between the two groups

There was no significant difference in hospitalization time, inflammation improvement time, scar score, first-stage healing and second-stage healing between the two groups (p > 0.05), as showed in Table 2.

**Table 2:** Comparison of postoperative healing between the two groups.

Index	Infected limited group (n = 50)	Inflammatory quiescent group (n = 50)	t/χ^2^	p-value
Length of stay (days)	15.16 ± 3.16	14.78 ± 2.78	0.638	0.525
Time for inflammation to improve (days)	4.01 ± 2.49	3.97 ± 2.14	0.086	0.932
Scar score (score)	3.78 ± 1.48	3.56 ± 1.39	0.766	0.445
Primary healing (example)	37	36	0.051	0.975
Second stage healing (example)	13	14	0.051	0.975

### Comparison of postoperative complication incidence and recurrence rate between the two groups

There was no significant difference in the incidence of postoperative complications between infection localized group and inflammatory quiescent group (2.00% vs. 0.00%, p > 0.05) and recurrence rate (1.00% vs. 2.00%, p > 0.05), as showed in Table 3.

**Table 3:** Comparison of postoperative complication incidence and recurrence rate between the two groups.

Grouping	Infected limited group (n = 50)	Inflammatory quiescent group (n = 50)	χ^2^	p-value
Number of recurrent cases	2	1		
Relapse rate	4	2	0.344	0.842
Auricular malformation	0	0		
Incision infection	1	0		
Incidence of complications (%)	2	0	1.01	0.604

## Discussion

In this study, the propensity matching score was used to compare the clinical efficacy of infection localized and inflammatory quiescent stage I surgery in the treatment of congenital preauricular fistula. It was found that for patients with congenital preauricular fistula in accordance with surgical indications, surgical incision should be designed reasonably.^12^^,^^13^ Active anti-infection can be performed in a limited period of infection, shorten the course of disease, reduce the psychological burden of patients and reduce their pain.

PSM involves calculating a propensity score for each study individual and matching individuals with similar covariates between the treatment and control groups to balance covariate differences and estimate treatment effects.^10^^,^^14^ Prior to PSM, there were significant differences in gender, age, Body Mass Index (BMI), and the number of patients with previous incision and drainage for infection between the two groups. However, after matching, there were no statistically significant differences in general characteristics between the two groups. Subsequent analyses were conducted based on this balanced dataset.

The study results showed that there was no statistically significant difference in the healing outcomes between the two groups, indicating that performing surgery during the infection-localized phase stage I did not result in significantly different healing outcomes compared to the inflammation-quiescent phase. Previous studies have also pointed out that there is no significant difference between the surgical treatment of stage I in the limited period of infection and the static period of inflammation, which is similar to the results of this study.^15^ This suggests that the timing of surgery does not significantly impact the healing process of congenital preauricular fistula. Further investigation into the occurrence of postoperative complications and recurrence rates in patients revealed no statistically significant difference between the two groups, indicating that performing surgery during the infection-localized stage does not increase the risk of complications or result in higher recurrence rates. These changes impede the drainage of secretions and result in poor local blood circulation. The patients require frequent incision and drainage of abscesses, outpatient dressing changes, and antibiotic use, prolonging the disease course and significantly impacting the patients' quality of life and financial burden.^16^ Therefore, for patients with congenital preauricular fistula, performing surgery during the infection-localized phase with a well-designed incision and active antimicrobial treatment can be beneficial.^17^ This approach not only shortens the disease course and alleviates the psychological burden on patients but also reduces their suffering.^18^

Traditional treatment methods typically choose the inflammation-quiescent stage, leading to prolonged suffering and non-resolution of the fistula tract in some patients, resulting in granulation tissue formation, fibroproliferation, and scar formation around the fistula tract.^19^ At the same time, our results also show that surgery in the limited period of infection does not increase the risk of complications or lead to a higher recurrence rate. Previous studies have also suggested that surgery in the limited period of infection is safer.^17^ It has important guiding significance for the treatment of congenital preauricular fistula.

When comparing the clinical efficacy of stage I surgical treatment of congenital preauricular fistula infection in the limited period and in the static period of inflammation, this study has the following limitations: First, the sample size may be limited, resulting in the statistical results being unstable and may not fully reflect the overall treatment effect. Secondly, differences in surgical procedures and postoperative care may affect efficacy evaluation, and further standardization of operating procedures is needed. In addition, factors such as individual patient differences and follow-up time may also introduce deviations. To solve these problems, it is recommended to expand the sample size, unify surgical and nursing standards, and extend follow-up time to improve the accuracy and reliability of the study results. At the same time, consideration can be given to introducing diversity factors during the analysis to more comprehensively evaluate the treatment effect under different conditions.

## Conclusion

In conclusion, the results of this study indicate that performing Stage I surgery during the infection-localized stage of congenital preauricular sinus yields clinical efficacy comparable to that of surgery during the inflammation-quiescent phase. Future research should further explore relevant treatment strategies and methods to provide more accurate and effective guidance for the treatment of congenital preauricular fistula.

## ORCID ID

Jibing Sun: 0009-0003-0596-7309

Tingting Wang: 0009-0001-9955-5609

Dong Chen: 0009-0008-5637-661X

Yanyan Mao: 0000-0002-5049-8965

Zhaomin Fan: 0000-0001-8966-4097

## CRediT authorship contribution statement

Jibing Sun-Data collection and analysis, drafting the manuscript; Tingting Wang, Dong Chen, Yanyan Mao, and Zhaomin Fan-Investigation; Yuechen Han-Conception, revising the manuscript. All authors have read and approved the final version of the manuscript.

## Consent for publication

Not applicable.

## Ethics and informed consent statement

Informed consent was waived for this retrospective study due to the exclusive use of de-identified patient data, which posed no potential harm or impact on patient care.

## Funding

This research was funded by 10.13039/501100001809National Natural Science Foundation of China (31700894).

## Data availability statement

All data generated or analyzed in this study are included in the present manuscript.

## Declaration of competing interest

The authors declare no conflicts of interest.
